# Motor Recovery and Synaptic Preservation after Ventral Root Avulsion and Repair with a Fibrin Sealant Derived from Snake Venom

**DOI:** 10.1371/journal.pone.0063260

**Published:** 2013-05-07

**Authors:** Roberta Barbizan, Mateus V. Castro, Antônio C. Rodrigues, Benedito Barraviera, Rui S. Ferreira, Alexandre L. R. Oliveira

**Affiliations:** 1 Laboratory of Nerve Regeneration, Department of Structural and Functional Biology, University of Campinas - UNICAMP, Anatomy, Campinas, Brazil; 2 FOB – USP, Bauru, Brazil; 3 CEVAP - Unesp, Botucatu, Brazil; University of Edinburgh, United Kingdom

## Abstract

**Background:**

Ventral root avulsion is an experimental model of proximal axonal injury at the central/peripheral nervous system interface that results in paralysis and poor clinical outcome after restorative surgery. Root reimplantation may decrease neuronal degeneration in such cases. We describe the use of a snake venom-derived fibrin sealant during surgical reconnection of avulsed roots at the spinal cord surface. The present work investigates the effects of this fibrin sealant on functional recovery, neuronal survival, synaptic plasticity, and glial reaction in the spinal motoneuron microenvironment after ventral root reimplantation.

**Methodology/Principal Findings:**

Female Lewis rats (7 weeks old) were subjected to VRA and root replantation. The animals were divided into two groups: 1) avulsion only and 2) replanted roots with fibrin sealant derived from snake venom. Post-surgical motor performance was evaluated using the CatWalk system twice a week for 12 weeks. The rats were sacrificed 12 weeks after surgery, and their lumbar intumescences were processed for motoneuron counting and immunohistochemistry (GFAP, Iba-1 and synaptophysin antisera). Array based qRT-PCR was used to evaluate gene regulation of several neurotrophic factors and receptors as well as inflammatory related molecules. The results indicated that the root reimplantation with fibrin sealant enhanced motor recovery, preserved the synaptic covering of the motoneurons and improved neuronal survival. The replanted group did not show significant changes in microglial response compared to VRA-only. However, the astroglial reaction was significantly reduced in this group.

**Conclusions/Significance:**

In conclusion, the present data suggest that the repair of avulsed roots with snake venom fibrin glue at the exact point of detachment results in neuroprotection and preservation of the synaptic network at the microenvironment of the lesioned motoneurons. Also such procedure reduced the astroglial reaction and increased mRNA levels to neurotrophins and anti-inflammatory cytokines that may in turn, contribute to improving recovery of motor function.

## Introduction

Upper limb movements as well as locomotion are accomplished through spinal motoneurons (MN), respectively located in the cervical and lumbar intumescences, resulting in the coordinated contraction of skeletal muscles. This coordination can be disrupted by lesions near the central/peripheral nervous system boundary. The trauma can interrupt the contact between a MN and the muscle fibers that it innervates [1]. Ventral root avulsion (VRA) has been used to model such disruptions. Within a few weeks of VRA, serious effects, including neuronal death, have been observed [2]–[4]. However, the surviving motorneurons have regenerative capacity [5].

VRA lesions are debilitating and are frequently associated with high-energy trauma such as motorbike accidents or complicated child-birth procedures [6]. In humans, these lesions cause proximal axonal injury at the CNS/PNS interface and result in paralysis and poor clinical outcome after restorative surgery [7]. Poor recovery of function following brachial plexus injury may be due to the substantial distances across which axons must regrow to reconnect with target muscles [8]–[10]. Also, traumatic lesions to the brachial plexus result in extensive motoneuron degeneration within the affected spinal cord segments, leading to muscular paralysis. The repair of such lesions is particularly difficult and uncertain due to the possibility of further spinal cord damage. To date, most surgical repairs have been restricted to the extra vertebral ruptured roots/fascicles. These structures may be reconnected through nerve autografting [11]. However, due to the variable and poor clinical outcome of this approach, the development of new repair strategies is needed. In particular, improvement of the methods are needed for replanting avulsed roots to the surface of the spinal cord.

The first attempts to repair avulsed roots were carried out in rats by Carlstedt et al. [12] and later by Cullheim et al. [13] in cats. In those seminal works, the avulsed roots were replanted to the lateral funiculus surface, demonstrating that motoneurons were capable of regrowing their ruptured axons and precisely directing those axons toward the PNS. The same technique has been applied to human subjects with limited success [1].

Other experimental attempts at root implantation have been performed using 9/0 non-resorbable sutures (Ethilon®) [14], tissue glue (Tisseel®) [14], [15], fibrin glue (TissueCol; Baxter B.V. Utrecht, the Netherlands) [8], and nerve grafts [16]. Due to the delicate nature of the spinal cord, the use of tissue glues may be advisable.

The fibrin glue has been used in neurosurgery for over then 20 years and does not induce damage to the nervous system [17]. The current study uses a fibrin sealant, developed by researchers from The Center for the Study of Venoms and Venomous Animals - CEVAP, in São Paulo State, Brazil. This particular adhesive fibrin sealant is produced without using human blood to avoid transmission of infectious diseases. A fraction of the gyroxin complex from the venom of *Crotalus durissus terrificus* is also used, instead of bovine thrombin. These substances mimic the final physiological steps in the coagulation cascade leading to clot formation. This fibrin sealant is also cheaper than commercial glues, easy to apply and is feasible for use in animal models. In this sense, it may be regarded as a potential alternative to treat CNS/PNS lesions.

By using the fibrin glue, the avulsed roots can be reconnected at the exact point of detachment, rather than ventrolaterally [14]. In addition, this experimental approach does not cause further lesions to the spinal cord or avulsed roots due to suturing [1] or the creation of small root implant pockets [8], [18]. In this study, we show that the reimplantation of avulsed roots at the lesion site leads to neuroprotection and results in significant motor recovery up to 12 weeks after surgical repair.

## Materials and Methods

### Experimental animals

Thirty adult female Lewis (LEW/HsdUnib) rats (7 weeks old) were obtained from the Multidisciplinary Center for Biological Investigation (CEMIB/UNICAMP) and housed under a 12-hour light/dark cycle with free access to food and water. The study was approved by the Institutional Committee for Ethics in Animal Experimentation (Committee for Ethics in Animal Use – Institute of Biology - CEUA/IB/UNICAMP, proc. n° 2073-1). All experiments were performed in accordance with the guidelines of the Brazilian College for Animal Experimentation. The animals were subjected to unilateral avulsion of the L4–L6 lumbar ventral roots (VRA) and divided into 2 groups: 1) VRA without reimplantation (VRA-Only, n = 15) and 2) VRA followed by lesioned root reimplantation (VRA+Implant, n = 15). In the second group, a fibrin-derived sealant was used at the site of avulsion. The peroneal functional index was calculated weekly for both groups of animals up to 12 weeks after injury. At this date, the animals were killed and the lumbar spinal cords were processed for immunohistochemistry (n = 5 for each group) and neuronal survival counting (n = 5 for each group). Additional animals were killed 4 weeks after injury and their lumbar spinal cords were processed for PCR (n = 5 for each group). Each animal's unlesioned, contralateral spinal cord side, served as an internal control.

### Ventral root avulsion (VRA)

The rats were anesthetized with 50 mg/Kg of ketamine (Fort Dodge) and 10 mg/Kg of xylazine (Köning) and subjected to unilateral avulsion of the lumbar ventral roots as previously described [19]–[20] (Figure S1 A). Unilateral avulsion was performed at the L4–L6 lumbar ventral root after unilateral laminectomy (right side). A longitudinal incision was made to open the dural sac, and the denticulate ligament was dissected. Finally, the ventral and dorsal roots were carefully separated so that the ventral roots associated with the lumbar intumescence could be identified and avulsed with fine forceps (No 4). After lesioning, the roots and spinal cord were returned to their original position, and the musculature, fascia and skin were sutured in layers. Chlorhydrate of tramadol was administrated by gavage after the surgical procedures (20 mg/kg) and 2.5 mg/day soluble in water during 5 days.

### Reimplantation of the motor roots

In the VRA+Implant group, the roots were replaced at the exact point of detachment, on the ventral surface of the lumbar spinal cord at the avulsion site with the aid of a fibrin sealant (Figure S1A). The sealant used herein is composed of three components as follows: component 1: cryoprecipitate rich in fibrinogen derived from bubaline blood; component 2: calcium chloride solution. Component 3: thrombin-like enriched solution. The appearance of the fibrin glue is shown in Figure S1B.

### Fibrin Sealant

The fibrin glue used in this study was provided by the Center for the Study of Venoms and Venomous Animals (CEVAP), UNESP. A thrombin-like fraction was isolated from crude *Crotalus durissus terrificus* snake venom through molecular exclusion and affinity chromatography [21]. The active fractions were then pooled and concentrated by dialysis [21]–[23]. The pool was analyzed for protein concentration [24] and characterized by sodium-dodecyl-sulfate polyacrylamide gel electrophoresis and immunoblotting (SDS-PAGE). Fibrinogen was obtained through wet cryoprecipitation of bubaline blood and contained an average of 1.2 mg/ml [23]. This material was stored at−20°C prior to use, at room temperature.

The fibrin glue, which is under patent, was composed of three separate solutions, as above mentioned, and homogenized immediately before use in a total final volume of 6 µl: 1: fibrinogen (3 µl), 2: calcium chloride (2 µl) and 3: a thrombin-like fraction (1 µl). During surgical repair of the avulsed roots, the first two components were applied and the avulsed roots were returned to their original sites. The third component was then added for polymerization. The reimplanted roots were then gently pulled from the spinal cord, and the stability of the fixation was observed to evaluate the success of the repair. If the reimplantation was not stable, more fibrin glue was added and the process was repeated.

### Specimen preparation

The animals were anaesthetized with an overdose of anaesthetic (mixture of xylasine and Ketamine,) and the vascular system was transcardially perfused with phosphate buffer 0.1 M (pH 7.4). For PCR, the rats were killed 4 weeks after VRA and their lumbar intumescences were frozen in liquid nitrogen for the PCR procedures. For neuron survival counting and immunohistochemistry the rats were killed 12 weeks after VRA and fixed by vascular perfusion of 10% formaldehyde in phosphate buffer (pH 7.4). The lumbar intumescence was dissected, post-fixed overnight and then washed in phosphate buffer and stored in sucrose (20%) for 8 hours before freezing. Transverse cryostat sections (12 µm thick) of spinal cords were obtained and transferred to gelatin-coated slides and dried at room temperature for 30 min before being stored at−20°C.

### Counting of motoneurons surviving two weeks after ventral root avulsion

Cell counts were performed on sections from the lumbar enlargement. Transverse cryostat sections of the spinal cords were stained for 3 min in aqueous 1% cresyl fast violet solution (Sigma-Aldrich, USA). The sections were then dehydrated and mounted with Entellan (Merck, USA).

The motoneurons were identified based on their morphology, size, and location in the ventral horn (dorsolateral lamina IX). Only cells with a visible nucleus and nucleolus were counted. Counts were made on 20 sections (every fourth section) along the lumbar enlargement on the ipsilateral and contralateral sides of each spinal cord. The absolute number of motoneurons on the lesioned and non-lesioned side of each section was used to calculate the percentage of surviving cells in each specimen. This percentage was calculated by dividing the number of motoneurons in the ipsilateral (lesioned) side by the number of neurons in the contralateral (non-lesioned) side and multiplying the result by 100. Abercrombie's formula [25] was used to correct for the duplicate counting of neurons: 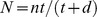 where *N* is the corrected number of counted neurons, *n* is the counted number of cells, *t* is the thickness of the sections (12 µm) and *d* is the average diameter of the cells. Because differences in cell size can significantly affect cell counts, the value of *d* was calculated specifically for each experimental group and for both ipsilateral and contralateral neurons. The diameters of 15 randomly chosen neurons from each group were chosen as a representative sample. These cells were measured in order to evaluate eventual changes in the neurons dimensions. (ImageTool software, version 3.00, The University of Texas Health Science Center in San Antonio, USA).

### Immunohistochemistry

Transverse sections of spinal cord were incubated with the following primary antibodies: mouse anti-synaptophysin (Dako, 1∶250), goat anti-GFAP (Dako, 1∶900), and rabbit anti-Iba1 (Wako, 1∶800). The primary antibodies were diluted in a solution containing 1% BSA in TBS-T (Tris-Buffered Saline and Tween). All sections were incubated overnight at 4°C in a moist chamber. After rinsing in TBS-T, the sections were incubated according to the primary host antibody (CY-3, Jackson Immunoresearch; 1∶250) for 45 minutes in a moist chamber at room temperature. The sections were then rinsed in TBS-T, mounted in a mixture of glycerol/PBS (3∶1) and observed with a Nikon eclipse TS100 inverted microscope (Nikon, Japan). For quantitative measurements, 3 representative images (with at least 2 MNs) of the spinal cord (L4–L6 at lamina IX, ventral horn) from each animal were captured at a final magnification of×200. Double blind quantification was performed in IMAGEJ software (version 1.33 u, National Institute of Health, USA) using the enhanced contrast and density slicing two features. The integrated density of pixels was systematically measured in six representative areas of the motor nucleus from each section, according to Oliveira et al. [26]. The integrated pixel density was calculated for each section of spinal cord, and then a mean value for each spinal cord was calculated. The data are represented as the mean ± standard error (SE).

### Functional Analysis

Motor function was analyzed using the peroneal functional index (PFI) and footprint pressure. For the gait recovery analysis, the CatWalk system (Noldus Inc., The Netherlands; http://www.noldus.com/animal-behavior-research/products/catwalk) was used. In the CatWalk system, the animal crosses a walkway with an illuminated glass floor. A high speed video camera Gevicam (GP-3360, USA) equipped with a wide-angle lens (8.5 mm, Fujicon Corp., China) is positioned underneath the walkway and the paw prints are automatically recorded and classified by the software as the animal moves across the walkway. The paw prints from each animal were obtained before and after the VRA. Post-operative CatWalk data were collected twice a week for 12 weeks.

PFI was calculated as the distance between the third toe and hind limb pads (print length) and the distance between the first and fifth toes (total toe spread). Measurements of these parameters were obtained from the right (lesioned) and left (unlesioned) paw prints, and the values were calculated using the following formula described by [27]–[28]. 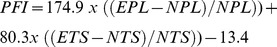 where N: normal, or non-operated side; E: experimental, or operated; PL: print length; TS: total toe spread, or distance between first to fifth toe.

The pressure exerted on the platform by individual paws was also evaluated. The Catwalk data from each day were expressed as an ipsi-/contralateral ratio.

### Real time polymerase chain reaction (PCR) super array

Total RNA was extracted from the ipsilateral and contralateral sides of the frozen lumbar intumescences, 4 weeks after avulsion, using the RNeasy Lipid Tissue Kit (cat n° 74804, Quiagen), according to the manufacturer's recommendations. The RNA was quantified using a NanoDrop Spectrophotometer (A260/280; model 2000, Thermo Scientific). The RNA (1 µg) obtained from five samples was reverse-transcribed using a commercial kit (AffinityScripts QPCR cDNA Synthesis Kit - Agilent Technologies, La Jolla, CA, USA) to achieve a final reaction volume of 20 µL. Real time quantitative PCR was performed on a Mx3005 P qPCR System (Agilent Technologies, La Jolla, CA, USA). The reactions were carried out with 12.5 µL of 2×SABiosciences's RT^2^ qPCR Master Mix, and 100 ng of cDNA template, in a final reaction volume of 25 µL. All quantifications were based on the Ct difference between the contralateral and ipsilateral side to the lesion, according to the kit manufacturer instructions and were normalized to the housekeeping gene Rplp1 (Ribosomal protein, large, P1). Samples obtained from five spinal cords from VRA-Only and VRA+Implant were analyzed using a real-time PCR array (RT^2^ Profiler™ PCR Array Rat Neurotrophins and Receptor, PARN-031, SuperArray Bioscience Corp., Frederick, MD, USA) containing the genes as shown in: http://www.sabiosciences.com/rt_pcr_product/HTML/PARN-031.

### Statistical analysis

Parametric data were analyzed using a one-way ANOVA and two-tailed Student's *t*-test. Nonparametric data were analyzed using a two-tailed Mann–Whitney U test. Functional analysis results were compared using one-way ANOVA and two-way ANOVA followed by Bonferroni *post hoc* test. The data are presented as the mean ± SE and the differences between groups were considered significant when the P-value was<0.05 (*),<0.01 (**) and<0.001 (***).

## Results

### Implanted roots protected spinal cord motoneurons after VRA

**Figure 1:**
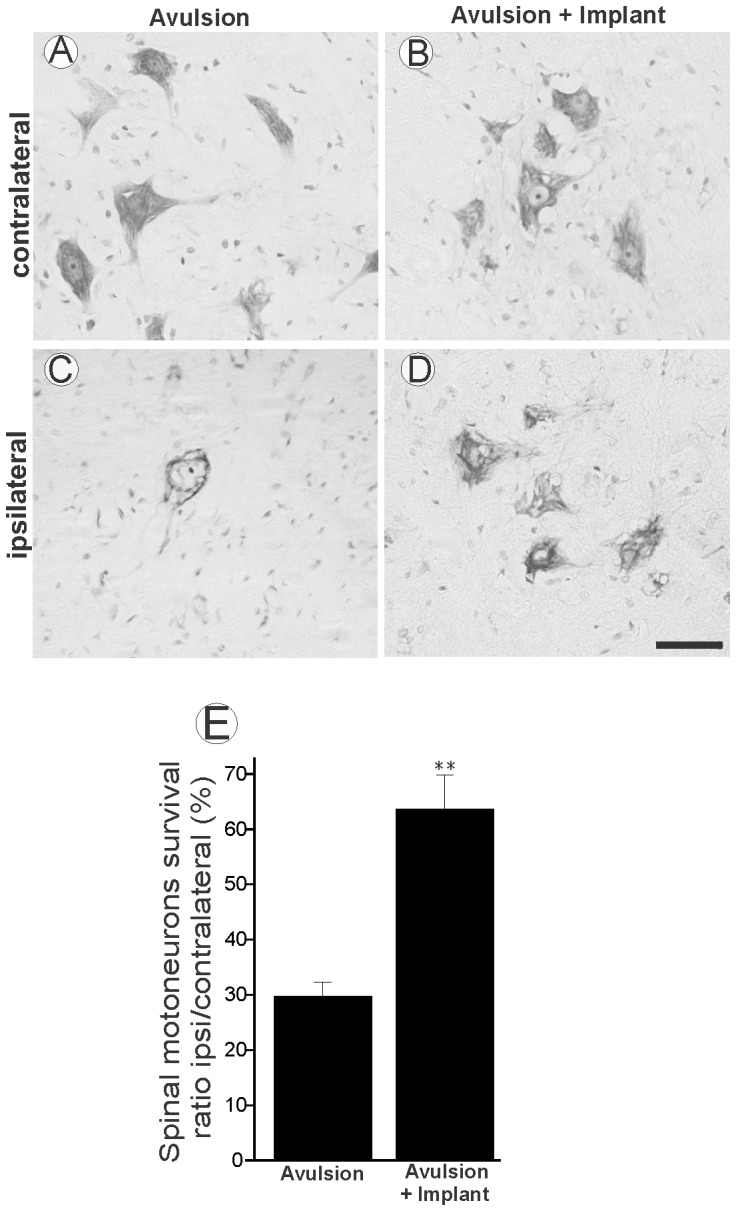
Neuroprotective effects of root reimplantation with fibrin sealant 12 weeks after VRA. (A and B) Motoneuron cell bodies of the side contralateral to the lesion, (C) Ipsilateral side of VRA-Only, and (D) VRA+Implant. Scale bar = 50 µm. (E) Percentage of neuronal survival after ventral root avulsion and implantation. Note a significant rescue of lesioned neurons in the implanted group. VRA-only was significantly different from implanted group (** p<0.01, n = 5).

The reimplantation of avulsed roots with fibrin sealant resulted in neuroprotection of proximally axotomized motoneurons, based on quantitative analysis of Nissl stained lumbar spinal cord sections (Figure 1). Neuronal survival was assessed as the ipsi-/contralateral ratio of motoneurons present in the lamina IX of the ventral horn. No significant differences between the numbers of motoneurons on the contralateral side in the different experimental conditions were observed. Figure 1E shows the ipsi-/contralateral ratio for the VRA-Only and VRA+Implant groups where a statistically significant neuroprotective effect in the implanted group was observed (VRA-Only 27.46%±4.34%; VRA+Implant 65.99%±9.05%, percentage of survival ipsi-/contralateral×100±SEM with p<0.01).

### Reduction of synaptic elimination and decreased glial reaction after VRA followed by implantation

**Figure 2:**
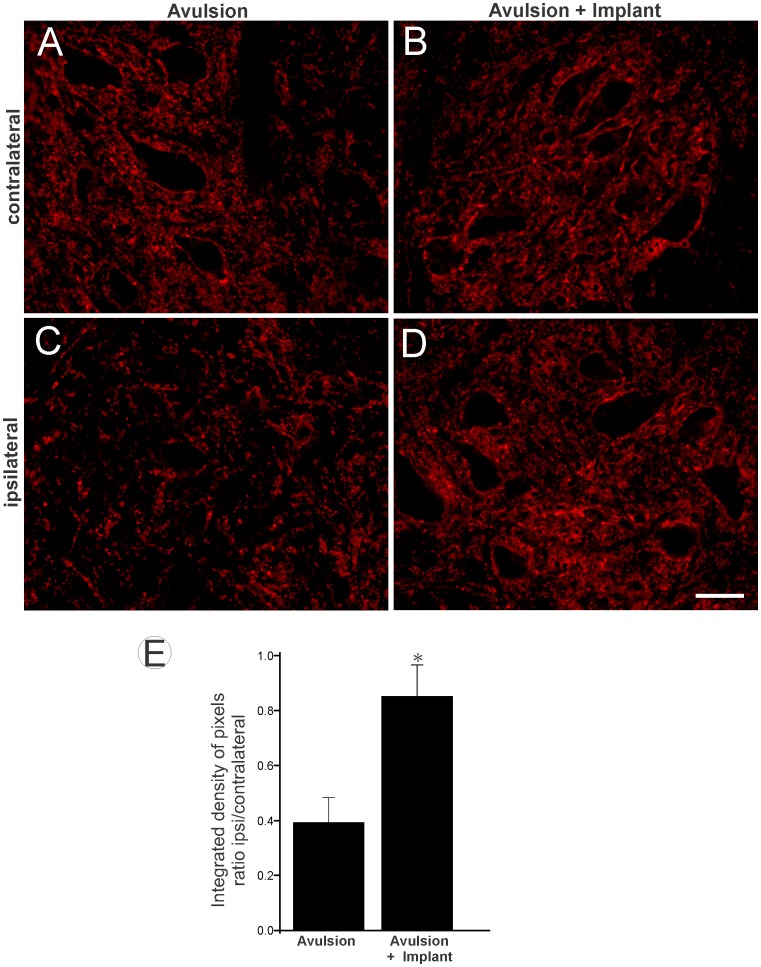
Synaptophysin immunolabeling in spinal cord ventral horn 12 weeks after VRA and reimplantation. (A and B) Normal synaptophysin immunoreactivity on the contralateral side to the lesion. (C) Ipsilateral side of the lesion in avulsed animals and (D) after root implantation. Scale bar = 50 µm. Observe the preservation of synaptophysin labeling at the surface of the lesioned motoneurons in the implanted group. (E) Quantification of immunolabeling. VRA-only was significantly different from the implanted group (* p<0.05, n = 5).

To evaluate changes in VRA synaptophysin labeling after implantation, we performed quantitative measurements of synaptophysin immunoreactivity in sciatic motor nuclei after avulsion (VRA-Only) and after avulsion followed by ventral root implantation (VRA+Implant). As shown in Figure 2, VRA-Only led to a significant decrease in synaptophysin expression. In contrast, in the VRA+Implant group, the repair resulted in preservation of synaptophysin immunoreactivity, especially in the immediate vicinity of the motoneurons. This finding indicates a decreased loss of inputs, putatively in apposition to the alpha motoneurons. These results also show that the preservation of inputs after reimplantation of avulsed roots is significantly different from VRA-Only (VRA-Only 0.39±0.08; VRA+Implant 0.85±0.11; mean ratio ipsi-/contralateral ±SE).

**Figure 3:**
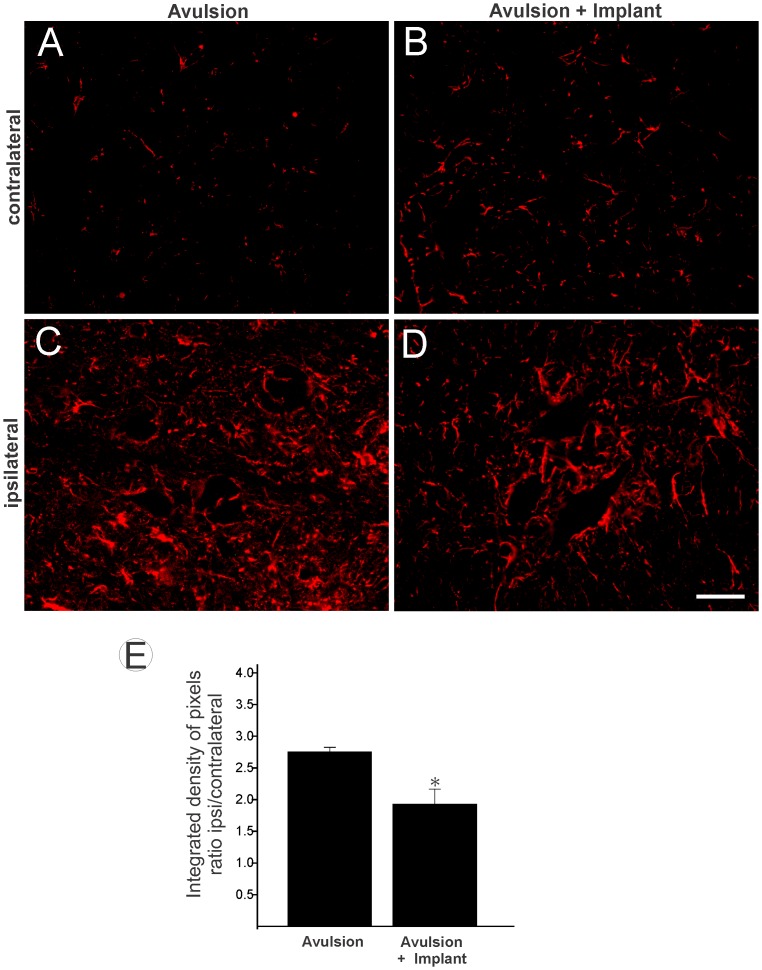
Glial fibrillary acidic protein (GFAP) immunolabeling in the spinal cord ventral horn 12 weeks after ventral root avulsion. (A and B) Normal GFAP immunolabeling on the side contralateral to the lesion. (C) Ipsilateral side of the lesion in avulsion and (D) Reimplantation after avulsion. Scale bar = 50 µm. Observe that ventral root implantation decreased astroglial reaction. (E) The mean ratio of the ipsi-/contralateral integrated intensity of pixels of the ipsilateral and contralateral sides in both groups. VRA-only was significantly different from implanted group (* p<0.05, n = 5).

Immunoreactivity against GFAP was used to analyze the degree of astroglial reactivity after lesion. Figure 3 shows spinal cord GFAP immunoreactivity contralateral to the lesion in (A) avulsed-Only and (B) VRA+Implant animals. This demonstrates the presence of GFAP-positive astrocytic processes in the microenvironment close to large motoneurons. On the side ipsilateral to the lesion, there was a significant increase in astrocyte reactivity after ventral root avulsion as demonstrated by the presence of reactive gliosis in the affected segments of the spinal cord. Increased GFAP labeling was particularly concentrated in the vicinity of the avulsed motoneurons, suggesting possible crosstalk between lesioned neurons and glia (Figure 3C). However, the VRA+Implant group displayed significant less intense astrogliosis in the ipsilateral side (Figure 3 D). (VRA-Only 2.75±0.07; VRA+Implant 1.93±0.25; mean ratio ipsi-/contralateral ±SE).

**Figure 4:**
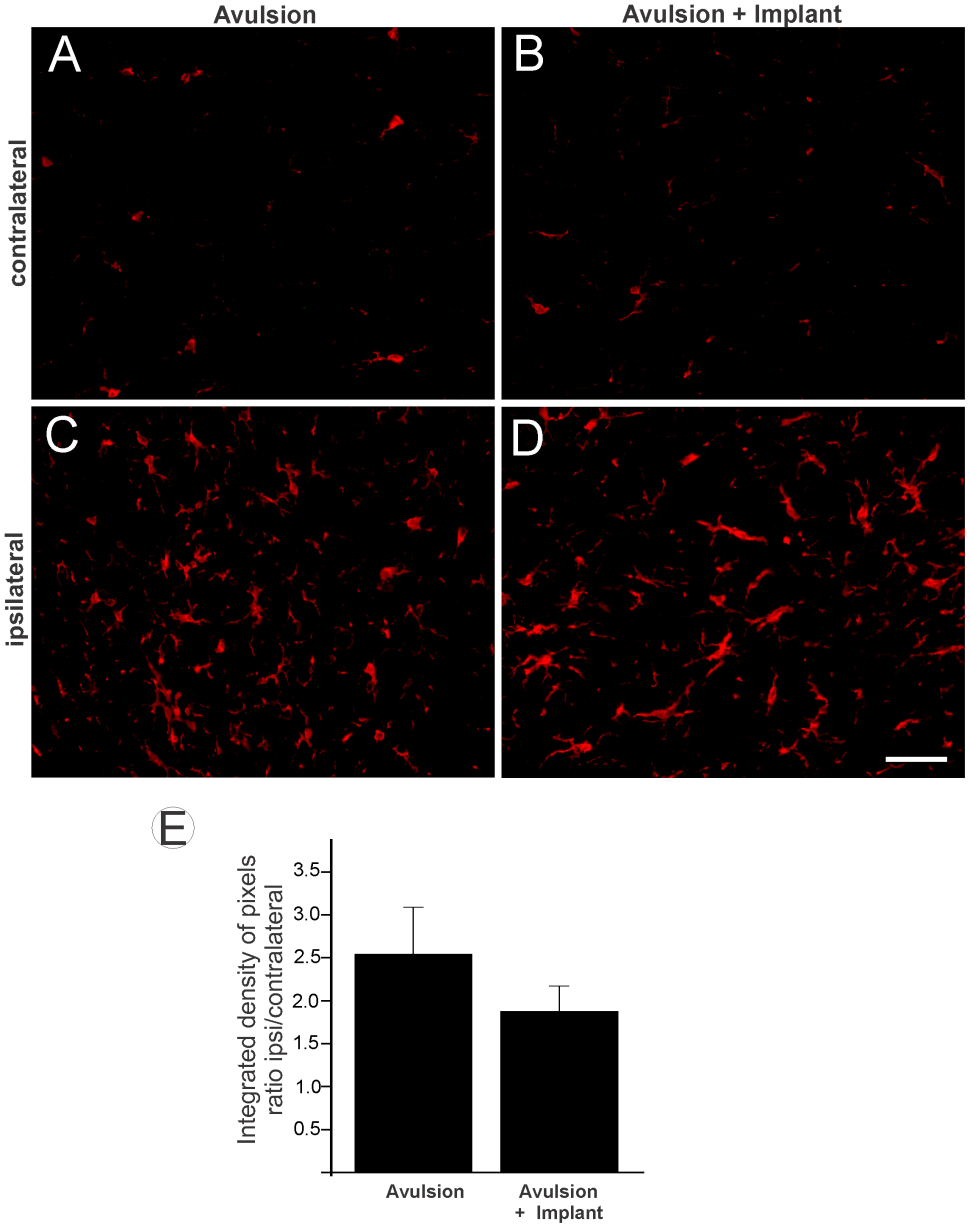
Iba1 immunolabeling in the spinal cord ventral horn. Immunohistochemical analysis of the anterior horn of the spinal cord was labeled with anti-Iba1 12 weeks after injury to assess the degree of microglial reactivity after root avulsion. (A and B) Normal immunolabeling of Iba1 on the contralateral side. (C) Ipsilateral side of the lesion in avulsion and (D) reimplantation after avulsion. Scale bar = 50 µm. (E) The mean ratio of the ipsil-/contralateral integrated intensity of pixels of the ipsilateral and contralateral sides in both groups. No significant differences between groups were observed (n = 5).

Iba-1 mmunoreactivity was used to assess the degree of microglial reactivity. The basal immunoreactivity on the contralateral side is presented in Figures 4A and 4B. The decrease in microglial reactivity in the VRA+Implant group (Figure 4D) was not significantly different from the VRA-Only group (Figure 4C) in the ipsilateral side analysis. (VRA-Only 2.54±0.74; VRA+Implant 1.87±0.29; mean ratio ipsi-/contralateral ±SE).

### Recovery of motor function by sealant implanted roots

**Figure 5:**
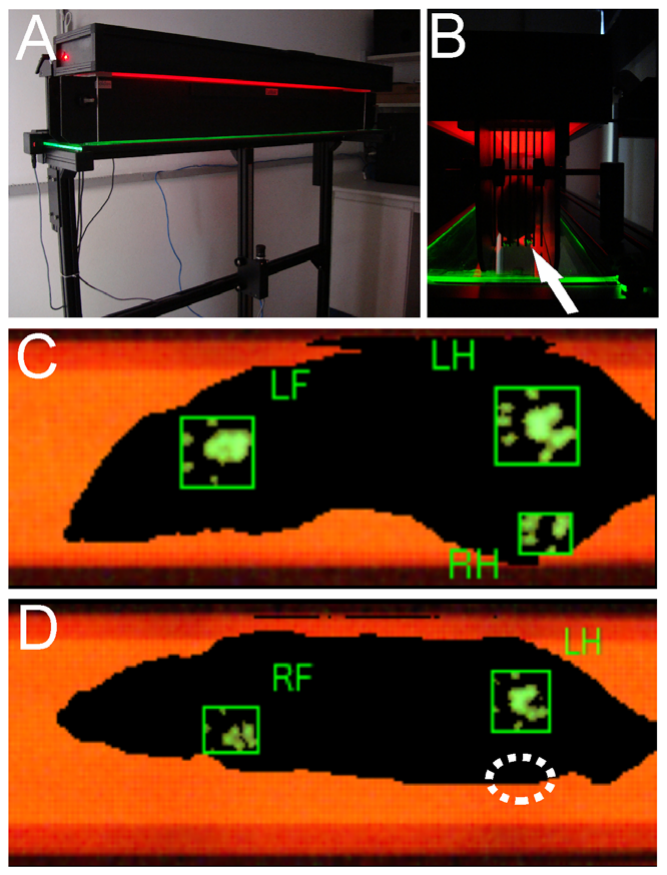
Paw pressure evaluation with the Catwalk system. The walking track test apparatus: (A) CatWalk machine and (B) an example of a rat at the walkway and the green plantar impression (arrow). In (C), observe a rat from the implanted group using the right paw 12 weeks after injury, whereas the avulsed rat without root repair cannot control the lesioned limb (D), dotted circle indicates the place where the paralyzed paw was supposed to be seen.

**Figure 6:**
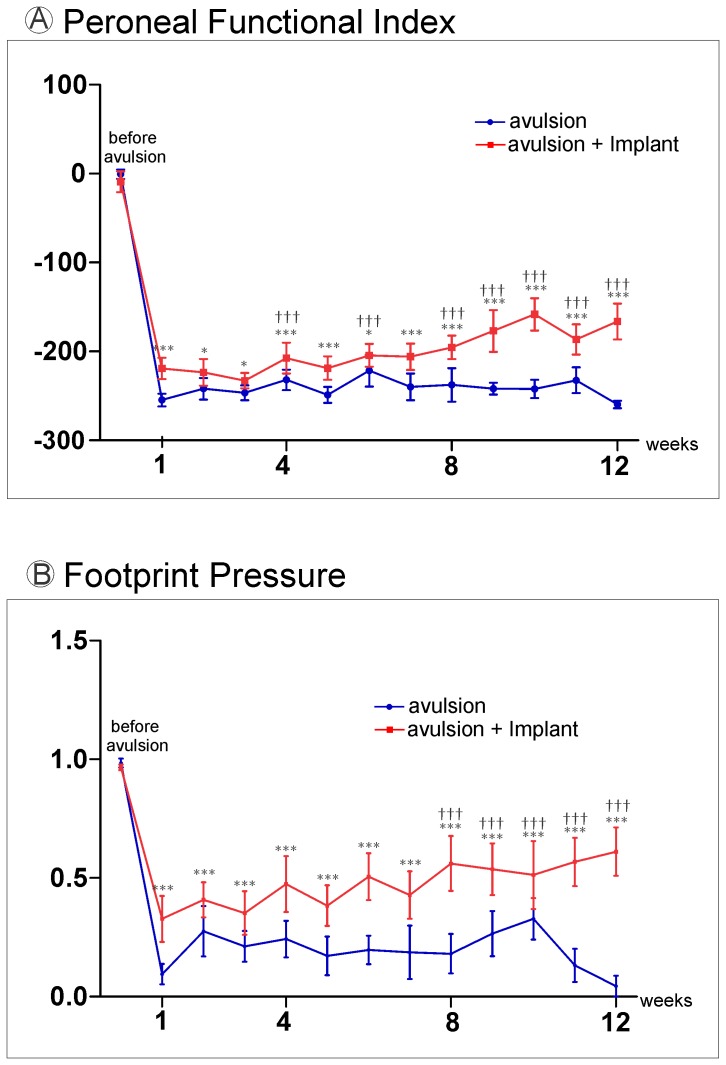
Motor function recovery following ventral root repair with fibrin sealant. Graph of the peroneal nerve functional index from one to 12 weeks after avulsion of the ventral roots (blue) and followed by avulsion reimplantation of the roots (red). (A) There is an improvement of motor performance in the fibrin sealant implanted group compared to VRA-only from the first week post lesion until the twelfth week (*** p<0.001 and * p<0.05, n = 10, two-way ANOVA followed by Bonferroni *post hoc* test). There is also an improvement of motor performance in the fibrin sealant implanted group from the first up to the twelfth week after injury (††† p<0.001 n = 10, one-way ANOVA followed by Bonferroni *post hoc* test). (B) Restoration of weight-bearing capacity following avulsion and reimplantation with the fibrin sealant. Values are expressed as the ratio of ipsi-/contralateral pressure exerted by the paw on the catwalk platform comparing fibrin sealant implanted group to VRA-only from the first week post lesion until the twelfth week (*** p<0.001; n = 10 two-way ANOVA followed by Bonferroni *post hoc* test). A significant restoration of weight-bearing capacity following avulsion and reimplantation with the fibrin sealant is also observed from the eighth week up to the twefth week, when compared to the first week after injury (††† p<0.001 n = 10, one-way ANOVA followed by Bonferroni *post hoc* test).

The recovery of motor function was assessed using the CatWalk System (Noldus Inc., The Netherlands) (Figure 5). Post-operative assessments of peroneal function were performed twice a week for 12 consecutive weeks. The preoperative peroneal functional index mean values (Figure 6A) did not significantly differ between groups. At the twelfth postoperative week, the VRA+Implant group presented with a significantly higher mean PFI (−147.65±4.60−mean±SE) compared to the VRA-Only group (−254.81±15.26) using the formula suggested by [27]. These results are consistent with the footprint paw pressure data (Figure 6B, Video S1 and Video S2) indicating that the VRA+Implant group was better able to support their body weight on the injured limb (VRA-Only 0.07±0.06; VRA+Implant 0.72±0.08; mean ratio ipsi-/contralateral±SE).

### Neurotrophic factors production by reimplantation

The real-time PCR super-array technique was used to investigate neurotrophin and receptor group differences. These results are presented in Table 1. Up- and down-regulation of neurotrophin and receptor genes in the different experimental conditions are presented as an ipsi-/contralateral ratio. Overall, it is possible to observe increased expression of neurotrophic factors and receptors following root reimplantation. In this sense, BDNF gene expression showed a 150% increase after reimplantation of the roots in comparison to avulsion only. In the same fashion, CNTF mRNA levels increased about 145% following the root repair procedure. GDNF levels were upregulated more than 250% in comparison to avulsion only, NGF increased about 140% and VGF about 200%. Most of the genes encoding neurotrophic receptors were also upregulated following root reimplantation. In this sense, CNTFr and NGFr mRNA increased three fold, and GDNF receptor (Gfra1) was upregulated by 150%.

**Table 1:** Fold Up- or down-regulation PCR ratio.

Gene Symbol	Avulsion	Avulsion+Implant	Function	Gene Symbol	Avulsion	Avulsion+Implant	Function
Adcyap1r1	−3.97	1.87	NTR	Il6r	−1.65	1.68	CY
Artn	1.06	1.47	NTR PNSD GFR	Il6st	1.73	5.10	CY
Bax	1.09	1.48	NG CP AP	Lif	1.13	3.23	CY IM
Bcl2	1.47	2.41	AP	Lifr	1.89	2.58	CY
Bdnf	2.08	3.18	NT DM GFR AP	LOC685671	1.68	2.79	TF
Cbln1	1.55	2.53	NG	Maged1	2.04	4.23	NTR
Cckar	−1.09	2.81	NR	Mc2r	1.39	3.14	NR
Cd40	−1.14	2.30	NTR AP IM	Mt3	−1.35	1.06	NTR DM
Cntf	1.58	2.28	NT CD	Myc	1.58	4.17	CP AP TR
Cntfr	−1.32	2.10	NT	Nell1	1.06	−1.33	NTR NG
Crh	1.72	2.19	NTR	Nf1	1.91	1.12	CD
Crhbp	−2.77	−1.12	NTR	Ngf	1.51	2.16	NTR PNSD
Crhr1	−1.02	2.55	NTR	Ngfr	2.77	4.56	TR PNSD GFR AP
Crhr2	1.22	2.17	NTR	Ngfrap1	−1.19	1.51	NTR AP
Cx3cr1	1.99	3.20	CY	Npffr2	1.11	2.75	NR
Cxcr4	2.71	2.38	CNSD CP CY	Npy	−1.39	1.13	NR
Fas	−1.22	3.01	NTR AP IM	Npy1r	1.39	3.56	NR
Fgf2	1.88	2.73	PNSD GFR CC CP CD	Npy2r	1.95	4.35	NR
Fgf9	1.04	3.03	PNSD GFR CC CP CD	Nr1i2	1.06	2.57	NTR
Fgfr1	1.07	4.14	CNSD	Nrg1	−1.23	1.56	NTR PNSD CD NR
Fos	1.91	3.48	NG NTR	Nrg2	−2.22	2.48	NTR PNSD NR
Frs2	1.67	1.53	NTR	Ntf3	−2.45	1.41	CNSD PNSD TR
Frs3	1.72	4.53	NTR	Ntf4	1.39	1.78	NTR NG
Fus	1.29	1.83	NTR	Ntrk1	−1.39	2.66	NTR NG CC
Galr1	1.42	2.17	NR	Ntrk2	−1.72	−1.01	NTR NG CC
Galr2	2.17	2.66	NR	Ppyr1	1.73	3.43	NR
Gdnf	1.23	3.16	NTR PNSD GFR	Pspn	1.64	2.91	NTR
Gfra1	4.08	6.28	NTR PNSD	Ptger2	1.18	3.12	NTR
Gfra2	1.61	1.18	NTR PNSD	Stat1	−1.16	2.06	NTR
Gfra3	1.45	1.95	NTR PNSD	Stat2	1.97	2.66	NTR
Gmfb	−1.12	−1.23	NTR	Stat3	1.47	2.93	CD IF NTR
Gmfg	1.02	2.13	NTR	Stat4	−1.46	2.04	CP NTR
Grpr	1.32	1.32	NR CP	Tacr1	1.89	1.39	NR
HcRt	−1.49	2.53	NTR	Tfg	1.39	1.32	NTR
Hcrtr1	2.81	2.01	NR	Tgfa	−1.00	1.26	CP
Hcrtr2	−1.46	2.13	NR	Tgfb1	1.01	1.68	GFR CC
Hspb1	−1.39	1.60	AP	Tgfb1i1	1.38	2.20	NTR IF
Il10	1.16	3.23	CP CY AP IM	Tp53	−1.16	2.51	CC CP CD AP NTR
Il10ra	1.12	4.47	CY	Ucn	1.80	2.66	NTR
Il1b	1.13	−1.41	CC CP IF	Vgf	1.24	2.66	NTR
Il1r1	−1.23	−1.10	CY	Zfp110	1.07	2.91	NTR
Il6	−1.61	1.13	CY AP IF	Zfp91	−18.90	3.34	NTR CD

RT-PCR results as the lesioned/non-lesioned ratio of genes in the lumbar spinal cord showing the fold change as up (+) or down (-) regulation in both groups.

Neurotrophins and Receptors = NTR,

Neuropeptides and Receptors  =  NR,

Neurogenesis  =  NG,

Central Nervous System Development  =  CNSD,

Peripheral Nervous System Development  =  PNSD,

Dendrite Morphogenesis  =  DM,

Growth Factors and Receptors  =  GFR,

Cell Cycle  =  CC,

Cell Proliferation  =  CP,

Cell Differentiation  =  CD,

Cytokines and Receptors  =  CY,

Apoptosis  =  AP,

Inflammatory Response  =  IF,

Immune Response  =  IM,

Transcription Factors and Regulators: TF.

The analysis of cytokines mRNA revealed an enhancement towards an anti-inflammatory profile following ventral root reimplantation. In this regard, a decrease of IL1b (25%) could be observed, together with upregulation of IL10 (278%) and IL10 receptor (∼400%)

Interestingly, Zfp91 gene showed a sharp downregulation following ventral root avulsion. Such decrease was totally reversed by root reimplantation. In fact, the reattachment of the roots results in upregulation of such gene, that is related to cell survival and suppression of apoptotic events.

## Discussion

A lesion at the CNS/PNS interface triggers extensive degeneration of spinal motor neurons [3], [29]–[30] and results in permanent loss of motor function. Following these lesions, a significant loss of motoneuron synapses occurs along with cell loss and other changes in the spinal cord microenvironment [31]–[34]. In humans, the brachial plexus is frequently affected by such severe injury [1], [35], leading to serious sequelae that decrease an individual's quality of life and ability to work.

Proximal axotomy can be experimentally reproduced after ventral root avulsion, leading to neuronal degeneration and spinal cord synaptic and glial changes. By repairing the avulsed roots, these changes can be reduced. Several techniques for replanting the roots lateral to the avulsion site have been used in the past. One of the novelties of the present work was to analyze motoneuron survival coupled with synaptic preservation after VRA and implantation at the site of injury. Also, the reimplantation of all avulsed roots was possible by using fibrin glue to stabilize the roots. This glue presents several advantages over the commercial counterparts since it is obtained from non-human blood, decreasing the possibility of disease transmission, and is cheaper to be produced. Nevertheless, it displays similar adhesion properties as compared to the currently commercial available products [36]. In the present study, this approach was used to successfully reduce neuronal death and to improve synaptic stability in the ventral horn of the spinal cord 12 weeks after VRA.

VRA is known to trigger severe degenerative processes in the cell bodies of injured neurons. These processes lead to death in approximately 80% of cells within two weeks of injury [2]. In a previous study, Wistar rats analyzed 16 weeks after VRA showed 27% motoneuron survival following avulsion and 53% cell survival when a commercial fibrin glue was used to replant the funiculus root laterally [8]. The authors suggested that implantation of avulsed ventral roots delays, but does not completely prevent, the degeneration of motoneurons, indicating that supplementary pharmacological approaches may be necessary. Hallin et al. [15] suggested that the survival of motoneurons after C4–C5 ventral root implantation in monkeys was related to the increased production of neurotrophic factors by the implanted ventral roots. They further suggested that a persisting defect in blood-brain barrier function at the site of the lesion allows trophic substances to access the injured area close to the motoneuron cell bodies [37]–[39].

Following an injury such as a proximal axotomy, which disrupts the contact between motoneurons and muscle fibers, there is a reduction of pre-synaptic terminals apposing to lesioned cells [40]. This may in turn block synaptic transmission in the acute period post-injury [32]–[34], probably also affecting nearby spinal segments that were not directly affected by the primary injury [41]. If reinnervation does not occur, such synaptic changes become irreversible [40]. Indeed, in the present study, these changes appear to be permanent twelve weeks after VRA injury, without root reimplantation (VRA-Only group). In our study, the VRA+Implant group showed preservation of synaptophysin immunolabeling, presumably reflecting the maintenance of spinal circuits related to the large axotomized motoneurons. This may in turn facilitate motor coordination recovery, once the regenerating motor axons reach their target muscle [13].

Following CNS lesions, a number of synaptic changes are observed in lamina IX, including activation of glial cells. Oliet et al. [42] have shown that astrocytes are able to reversibly retract synapses following nervous system damage, showing that they are capable of responding to changes in the microenvironment [43]. These effects may be related to the hypertrophy of astrocytic processes around the injured motoneuron cell bodies, where dense networks that inhibit and limit axonal growth, sprouting and regeneration form [44].The VRA+Implant group displayed a decrease in GFAP expression. This decrease may have contributed to the higher neuronal survival and increased synaptic stability observed in the present study. Under normal conditions, astrocytes synthesize extracellular matrix proteins, adhesion molecules, and trophic factors [43]. Also, such cells form the blood brain barrier, regulate extracellular ionic buffering and control neurotransmitter uptake [43]. However, following lesion to the CNS, astrocytes may be actively involved in the displacement of presynaptic terminals, contributing to synapse network degeneration [45]. Furthermore, reactive astrocytes produce pro-inflammatory cytokines, as well as chondroitin sulfate rich extracellular matrix that hampers axonal growth [46]–[48], the reversal of which may promote neuronal survival. Overall, the gene array data herein provide evidence that VRA leads to such pro-inflammatory scenario, which may be connected to the increased astrogliosis. In contrast to the observed reduction in astrogliosis, reimplantation of ventral roots did not significantly alter microglial reactivity. This observation may reflect the 12-week post-surgery survival time. It is possible that microglial reactivity may be altered on a shorter time scale. The peak of microglial reactivity is estimated to occur within the first two weeks following injury. During that critical window, microglial cells may develop opposite functions as neuroprotective producers of neurotrophic molecules [49], [50] or may contribute to a further neurodegeneration, by developing phagocytic characteristics and secreting toxic substances such as nitric oxide and superoxide [50]–[51].

While the activity of late microglia may be related to neuroprotection, astrocytes are generally believed to play a non regenerative role, due to the secretion of various molecules that inhibit axonal growth [52].

Penas et al. [53] performed ventral and dorsal root avulsion and observed an increase in neuroprotection following treatment with Pre084 and a decrease in astroglial immunoreactivity after three weeks. They did not observe changes in microglia immunoreactivity, suggesting that microglial function is not essential to reduce the degeneration of MNs. Furthermore, Freria et al. [20] showed no changes in microglial reactivity in G-CSF treatment two weeks after VRA. They also observed synaptic and MN preservation with increased astroglial immunoreactivity and neuronal survival.

Ohlson et al [54] did not observe differences in astroglial and microglial reaction, and macrophage influx, when comparing avulsed and implanted groups, four weeks after lumbosacral ventral root avulsion injury. They have suggested that the implant itself may compensate the possible negative effects of gliosis and inflammation. However, when analyzing the dorsal horn after VRA, in order to study the occurrence of neuropathic pain, Bigbee et. al. [55] observed increased microglial activity 8 weeks after avulsion in comparison with the implanted group [55]. In this way, different experimental conditions and survival times have provided apparently conflicting results, indicating the need of caution when interpreting the role of glial reaction following VRA and root reimplantation.

In the present study, we did not specifically analyze the microglial cells subset. However, RT-PCR results demonstrated the presence of possible M2 profiles, which are neuroprotective and known to support long-distance axonal growth [56]. We observed significantly more IL-10, IL-10 receptor and IL-6st mRNA in the VRA+Implant group. Additionally, IL-6, which promotes neuronal survival during excitotoxicity events, was upregulated after root reimplantation [57], [58].

The microarray method is effective to provide an overall scenario regarding gene regulation, although caution is necessary in order to avoid misinterpretation of data [59]. In this regard, the results herein have taken into account the Ct difference between the contralateral and ipsilateral side to the lesion as well as the Ct of the endogenous gene (Rplp1), according to the kit manufacturer instructions. Nevertheless, it is not possible to conclude that changes in mRNA levels are particularly associated with glia, neurons or infiltrating cells.

Since most of the molecular changes that result in improved neuronal survival and axonal growth occur during the first weeks post injury, the present investigation used this technique at such early stage. Motor function and neuronal survival estimation were rather performed at a later survival time (12 weeks), in order to assure that they were enduring.

Several molecules and substances are known to protect nervous system cells and to contribute to tissue regeneration. For example, heat shock proteins have been shown to rescue motoneurons after axotomy [60]. The present data indicate that upregulation of chaperone Hspb1 reinforces the positive effects of root repair at the lesion site. Increased VGF expression in the implanted group was also observed. VGF has been shown to be neuroprotective in an ALS mouse model [61].

Hallin et al. [15] suggested that the correction of motor deficits after ventral root avulsion and reimplantation in monkeys depends on the initial stimulation of CNS/PNS regeneration by neurotrophic factors and receptor expression, as well as the presence of extracellular matrix molecules, including collagen type I, III and IV and laminin [62]. Consistent with this hypothesis, the present study demonstrated an increase in neurotrophins in the root-repaired specimens, including BDNF, NT-3 and GDNF. Such neurotrophic molecules are directly related to axonal sprouting and elongation after peripheral nerve injury and during development [63]. These neurotrophic molecules have also been linked to motor recovery after cerebral ischemia [64]. The ciliary neurotrophic factor (CNTF) and its receptor (CNTFr) were also up-regulated in the VRA+Implant group. The potent neuroprotective action of both molecules has been demonstrated in different situations, including peripheral axotomy in neonatal animals and in cell culture [65].

One important finding herein is the high upregularion of the Zinc Finger Protein 91 gene (ZFP91), following root reimplantation. The gene product of ZFP91 probable functions as a transcription factor, allowing cell growth and suppressing apoptosis [66].

A complete understanding of the regeneration process following the root repair also requires the behavioral evaluation of gait recovery. This is because neuronal survival alone may not result in functional recovery. In this sense, the use of a more refined system to investigate locomotion may provide important data. In the present work we have used the CatWalk system that provides precise measurements of the paw prints as well as quantitative information regarding paw pressure.

Interestingly, root reimplantation led to a less severe decrease of the peroneal functional index already from the first week, when the target muscles were still certainly denervated. This may reflect a better preservation of the microenvironment surrounding the lesioned motoneurons as well as the spinal segments above and below the injury. The reduction of inflammation and glial reaction may contribute to this process as suggested elsewhere [41]. In line with this, the improvement of foot print pressure was a hallmark of the reimplanted group and reinforced the functional index findings. Again, the acute loss of function was less intense with the root repair, indicating that other muscle groups were more functional in comparison to the avulsion alone group, and partially compensated the posture loss following injury. This is probable since femoral (L3–L4) and obturator (L2–L4) nerves are partially preserved following L4–L6 avulsion [67].

Altogether, the histological, molecular and functional observations of the current study indicate that the root reimplantation performed with support of fibrin glue was neuroprotective, enhancing motoneuron survival and improving motor function recovery by facilitating the regeneration process. Whether or not this is due to the venom remains to be confirmed because as the results stand it is not clear whether it is the replant site or the glue which may be responsible.

## Supporting Information

Figure S1 **Ventral root implant procedures: (A) Rat lumbar intumescence in a lateral/dorsal view.** After L4–L6 ventral avulsion the roots were replanted with the sealant at the avulsioned site. * represents the dorsal roots that were carefully spared in order to allow the ventral root avulsion procedure. Scale bar = 1 mm. (B) The Fibrin Sealant network after polymerization inside a glass tube.
(TIF)

Video S1 **Walking track test video showing an example of the gait pattern in an animal from the avulsion-only group, 12 weeks after surgery.**
(WMV)

Video S2 **Walking track test video showing an example of the gait pattern in an animal from the avulsion+implant group, 12 weeks after surgery.**
(AVI)
