# Supplementary figures and images for: Motor Recovery and Synaptic Preservation after Ventral Root Avulsion and Repair with a Fibrin Sealant Derived from Snake Venom
Source: PLoS One. 2013 May 7;8(5):e63260. doi: 10.1371/journal.pone.0063260 (PMC3646764; doi:10.1371/journal.pone.0063260)

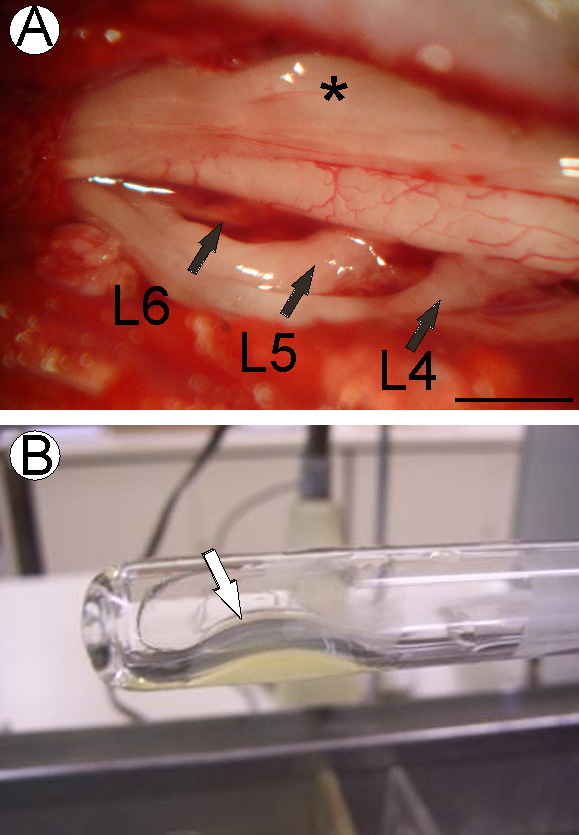

Supplement: Figure S1 — Ventral root implant procedures: (A) Rat lumbar intumescence in a lateral/dorsal view. After L4–L6 ventral avulsion the roots were replanted with the sealant at the avulsioned site. * represents the dorsal roots that were carefully spared in order to allow the ventral root avulsion procedure. Scale bar = 1 mm. (B) The Fibrin Sealant network after polymerization inside a glass tube. (TIF) [file pone.0063260.s001.tif]
